# When actionable genomic findings do not translate into treatment in precision oncology

**DOI:** 10.1093/jncics/pkag115

**Published:** 2026-10-16

**Authors:** Ewan K Cobran

**Affiliations:** Department of Quantitative Health Science, College of Medicine and Science, Mayo Clinic, Scottsdale, AZ, United States

## Abstract

Comprehensive genomic profiling has expanded the ability to identify potentially actionable molecular alterations across cancers, but identification of an actionable finding does not ensure that a patient will receive a genomically matched therapy. Studies of precision-oncology programs and molecular tumor boards (MTBs) consistently demonstrate substantial attrition between treatment recommendation and treatment initiation. The reasons extend beyond molecular interpretation and include the timing of genomic profiling, clinical deterioration, geographic availability of biomarker-selected trials, insurance and financial barriers, and disparities in access to specialty cancer care. Importantly, these barriers arise after a potentially useful molecular finding has already been identified, creating a distinction between molecular actionability and practical treatment access. This commentary argues that the next implementation challenge for precision oncology is to make clinically meaningful genomic findings realistically actionable. Comprehensive genomic profiling should occur early enough to inform treatment planning; MTB recommendations should be linked to realistic treatment and trial opportunities; financial and insurance barriers should be anticipated when recommendations are made; and equity should be evaluated beyond access to testing to include receipt of genomically informed treatment. As genomic profiling becomes increasingly integrated into routine cancer care, precision oncology should be judged not only by its ability to identify therapeutic opportunities but also by whether patients can reach those opportunities while they remain clinically relevant.

## Actionability is only the beginning

Precision oncology seeks to tailor cancer treatment using molecular characteristics of an individual patient’s tumor together with clinical features that influence therapeutic choice. Comprehensive genomic profiling can identify genomic alterations associated with sensitivity or resistance to therapy, approved targeted treatments, tumor-agnostic therapies, and eligibility for biomarker-selected clinical trials.^1-3^ Molecular tumor boards (MTBs) are multidisciplinary groups that integrate genomic findings with pathology, clinical oncology, pharmacology, genetics, bioinformatics, and available evidence to help translate complex molecular results into clinically relevant recommendations.^4^^,^^5^

Yet an important distinction remains between identifying an actionable alteration and delivering treatment based on that alteration. Much of the precision-oncology literature has focused on whether genomic profiling identifies actionable alterations and whether molecular matching is associated with clinical benefit. A distinct implementation question is what happens after an actionable finding has been identified and a treatment recommendation has been made. The magnitude of this downstream gap is evident across precision-oncology studies. MOSCATO 01 enrolled adults with hard-to-treat advanced cancers; among 843 patients with successful molecular profiling, 411 had an actionable alteration and 199 received genomically matched targeted therapy.^6^ Among evaluable treated patients, 33% achieved a progression-free-survival ratio >1.3 relative to the preceding therapy, while the objective response rate was 11.6%. In WINTHER, 303 patients with advanced cancers consented to DNA- or RNA-guided profiling, 107 received treatment consistent with study recommendations, and 26.2% achieved durable stable disease or an objective response.^7^ I-PREDICT used tumor profiling to construct individualized, often multidrug, regimens for patients with advanced cancers; 49% of consented patients received personalized therapy, and higher molecular matching scores were associated with better disease control and longer progression-free and overall survival.^8^ These studies demonstrate clinical benefit in subsets of patients while also showing that identifying a molecular opportunity does not ensure that a patient will receive, or benefit from, a matched therapy.

Real-world MTB experience reveals similar patterns. A regional precision-medicine MTB identified at least 1 clinically actionable nonstandard-of-care alteration in 69% of evaluated patients, but many recommendations were not pursued because patients were responding to existing treatment, died before treatment could be initiated, experienced rapid performance-status deterioration, lacked financial support, or could not access the recommended clinical trial geographically.^9^ A systematic review of 14 MTB studies involving 3328 patients likewise identified insurance, geographic location of trials, patient or physician decisions, and the need to complete standard-of-care treatment as recurrent reasons MTB recommendations were not implemented.^5^

A recent single-center analysis further illustrates both the implementation gap and the distinction between treatment receipt and treatment benefit. Of 582 patients evaluated by an MTB, 324 received targeted-therapy recommendations and at least 76 subsequently received the proposed treatment.^10^ Among 31 treated patients with paired progression-free-survival data, 54.8% achieved a PFS2/PFS1 ratio of at least 1.3; in a separate survival analysis among previously treated patients, receipt of the MTB-recommended therapy was associated with longer overall survival, although the observational design precludes causal inference.^10^ These findings show that some patients derive meaningful benefit after implementation while reinforcing that an MTB recommendation, and even receipt of matched treatment, should not be equated with guaranteed clinical benefit. The field has developed considerable capacity to find molecular treatment opportunities. It now needs comparable capacity to ensure that those opportunities remain clinically relevant and realistically obtainable.

## An actionable finding is not necessarily an immediate treatment

The term actionable can imply a direct transition from molecular finding to therapy. Clinically, however, actionability exists along a spectrum. Some genomic alterations are supported by regulatory approval and prospective evidence within a specific tumor type. Others identify therapies supported by evidence from another cancer, small studies, case series, or early-phase trials. The European Society for Medical Oncology Scale for Clinical Actionability of molecular Targets was developed specifically to distinguish these different levels of evidence.^11^ This distinction matters because a molecular target is not equivalent to an indication for immediate treatment. A patient may appropriately continue an effective standard therapy despite having an actionable alteration. A targeted therapy may become relevant only at progression or after established treatment options have been exhausted. In other circumstances, a molecularly suggested therapy may have less compelling evidence than the available standard treatment.

The Australian Molecular Screening and Therapeutics program provides contemporary evidence for the importance of evidence strength. In patients with advanced rare or refractory solid tumors, genomic matching was associated with observable differences in overall survival when the biomarker–treatment relationship was supported by prospective clinical-trial evidence, whereas weaker evidence tiers were less consistently associated with benefit.^12^ The findings reinforce that the clinical value of actionability depends not simply on finding a target but on the quality of evidence connecting that target to a treatment in the patient’s clinical context.

Molecular tumor boards, therefore, serve an important role not only by finding treatment options but also by prioritizing them. Reviews of MTB practice emphasize that recommendations must integrate genomic findings with tumor type, prior therapies, disease trajectory, evidence strength, available trials, and access to treatment.^4^ The practical question is not merely whether a finding is actionable. It is whether it is actionable for this patient at this point in the disease course. Treatment implementation and treatment effectiveness are also distinct dimensions of precision oncology. Receipt of a genomically matched therapy does not ensure clinical benefit, just as nonimplementation does not necessarily indicate failure. A patient may appropriately remain on an effective standard therapy, may no longer be clinically eligible for the recommended treatment, or may have an alternative treatment supported by stronger evidence. Implementation metrics should therefore distinguish potentially avoidable failures to reach an appropriate matched therapy from clinically appropriate decisions not to initiate one.

## Timing of comprehensive genomic profiling can determine whether a finding is usable

One of the most consequential barriers arises before a treatment recommendation is ever made: genomic information may become available too late. Historically, many precision-oncology programs enrolled heavily pretreated patients with refractory advanced cancers. In MOSCATO 01, patients receiving genomically matched treatment had received a median of 4 prior treatment lines.^6^

Participants in WINTHER had received a median of 3 previous therapies.^7^ These studies were critical demonstrations of precision oncology, but they also illustrate the narrow clinical window within which genomic information has often been introduced. In WINTHER, deterioration in health or death before treatment initiation contributed substantially to patient attrition, leading the investigators to suggest that future studies consider enrollment earlier in the disease course.^7^ The consequences of late testing are clinically important. Patients with advanced cancer may experience deterioration in performance status during the interval required for tissue acquisition, molecular profiling, MTB review, trial screening, drug access, and treatment initiation.^9^^,^^13^^,^^14^ An actionable result may therefore become available when a patient is no longer sufficiently well to receive the therapy it identifies.^9^^,^^13^^,^^14^

In the regional MTB experience, 20.3% of patients who did not pursue a nonstandard recommendation died before the recommended treatment could be started or considered, and another 8.5% experienced rapid deterioration in performance status.^9^ In I-PREDICT, investigators specifically identified deterioration and death of patients with end-stage disease before treatment implementation as contributors to low matching rates observed in precision-oncology studies.^8^ WINTHER similarly found that better performance status and fewer previous therapies were associated with longer progression-free survival.^7^ These observations support a straightforward clinical principle, molecular information must be generated while there is still time to use it.

Earlier comprehensive genomic profiling does not necessarily mean earlier targeted treatment. Rather, it creates time to anticipate subsequent treatment decisions. An actionable alteration identified while a patient is benefiting from current therapy can be documented for a potential future line of treatment, reviewed by an MTB, and linked to relevant clinical trials, allowing therapeutic options to be considered before disease progression creates greater clinical urgency.^2^^,^^4^^,^^13^

Earlier testing may also provide time to address practical barriers that can be difficult to resolve once the therapeutic window narrows, including confirmatory testing, specialist or institutional referral, insurance authorization, clinical-trial screening, and travel or transportation arrangements.^15-17^ Precision oncology therefore needs to move from late molecular rescue toward prospective molecular treatment planning. Earlier treatment planning also depends on generating evidence for targeted therapies before patients reach heavily pretreated, late-stage disease. The US Food and Drug Administration Oncology Center of Excellence’s Project FrontRunner encourages sponsors to consider developing and seeking approval for oncology therapies in earlier treatment settings rather than routinely beginning development only after numerous prior lines of therapy or exhaustion of available options.^18^ Such efforts complement earlier genomic profiling by increasing opportunities to evaluate matched treatments while patients have better performance status and a wider clinical window in which therapy can be delivered.

The appropriate timing will differ by cancer and disease stage. But when genomic information is likely to influence treatment during advanced disease, waiting until conventional options have been exhausted can substantially reduce the probability that an actionable result will ever become an actionable treatment.

## Genomic actionability can change during the disease course

Earlier profiling does not eliminate the potential value of reassessment at disease progression. Cancers evolve under natural selection and therapeutic pressure, and treatment can change the relative abundance of pre-existing clones while selecting resistant subclones.^19^^,^^20^

Consequently, a genomic profile obtained earlier in the disease course may not fully represent the therapeutically relevant molecular landscape of relapsed or progressive disease. Acquired resistance alterations or newly dominant molecular targets may therefore change the relevance of an earlier MTB recommendation.

When the result could meaningfully alter management, repeat molecular profiling at progression can be considered in the context of tumor type, prior therapy, availability of adequate tissue, expected clinical utility, and the patient’s condition. American Society of Clinical Oncology (ASCO) has noted that repeat genomic testing may be useful after acquired resistance to targeted therapy when known resistance mechanisms can influence next-line treatment selection.^19^ Repeat tissue biopsy may not always be feasible, and circulating tumor DNA can provide a less invasive complementary approach in selected settings, although sensitivity and interpretation vary by tumor type and disease burden. Molecular tumor boards can help determine when repeat profiling is likely to add actionable information and reassess earlier recommendations considering tumor evolution. Precision oncology therefore requires both timely initial profiling and recognition that molecular actionability may change across the disease course.

## Clinical-trial geography can turn molecular actionability into theoretical actionability

For many actionable genomic alterations, access to a matched therapy may depend on participation in a clinical trial rather than availability of an approved treatment. This creates a second translational challenge. A genomic report may identify a trial for which a patient appears molecularly eligible, yet molecular matching does not ensure actual eligibility or access.^21^ The trial may be available only at a distant academic center, require substantial or repeated travel, impose additional clinical eligibility criteria, or have limited enrollment availability.^13^^,^^22^ Consequently, a molecular trial match does not necessarily represent a geographically or practically accessible treatment opportunity.^13^

The scale of geographic variation in cancer-trial access is substantial. A recent national analysis identified 6710 US cancer treatment trials open across 1836 sites in 2022.^23^ Further, 70% of US counties had no active cancer treatment trial, including 86% of nonmetropolitan counties. Approximately 26% of adults aged 55 years or older lived more than a 1-h drive from a site offering at least 100 trials.^23^ These geographic constraints become especially important in precision oncology because uncommon genomic alterations can sharply narrow the number of relevant studies. Real-world MTB experience demonstrates the consequences. In the regional MTB study, 10.2% of patients who did not pursue a recommendation received another treatment specifically because the recommended clinical trial was geographically inaccessible.^5^

An academic-community MTB initiative in the rural southeastern United States provides an even more direct example.^21^ Among 151 patients, 87 had actionable genomic biomarkers and 27 were matched to a clinical trial, yet only one of the 27 patients matched to a trial enrolled. Community oncologists identified distance to the trial center, transportation, and lack of local trials as major barriers.^21^ This distinction deserves greater attention in molecular reporting and MTB recommendations.

A clinical trial should not be considered actionable solely because a patient’s molecular alteration appears among its eligibility criteria. Actionability also depends on whether the trial is actively recruiting, whether the patient meets key clinical eligibility requirements, and whether participation is geographically and logistically feasible.^17^^,^^24^^,^^25^ Precision-oncology programs should therefore integrate realistic trial navigation with molecular interpretation.^25^

Recommendations should distinguish a molecular trial match from an accessible trial opportunity by incorporating recruitment status, clinical eligibility, trial location, and other barriers to participation.^9^^,^^17^^,^^24^ When referral to another institution is required, navigation should ideally begin when the recommendation is made rather than leaving patients and referring oncologists to identify and negotiate access independently.^9^^,^^21^^,^^25^

Decentralized and hybrid clinical-trial models may help reduce some geographic barriers by shifting selected trial activities away from a single central research site. Depending on the protocol and investigational therapy, remote consent, telemedicine visits, local laboratory or imaging assessments, home-based procedures, and coordination with community clinicians can reduce the frequency and duration of travel required for participation.^26^ Satellite trial sites, community-based research networks, and academic-community partnerships offer complementary approaches by bringing research infrastructure closer to patients. These strategies cannot eliminate all barriers, particularly when investigational treatment or specialized monitoring must occur at a central site, but they can make a molecular trial match more realistically accessible.^26^ Finding the right trial is only useful if the patient can reach it.

## Financial toxicity and treatment access after an actionable finding

Even when a genomically matched therapy exists, financial access can determine whether molecular actionability becomes treatment. Financial toxicity refers to the material and psychosocial burden associated with the costs of cancer care, including direct out-of-pocket expenses and indirect costs such as travel, lost employment, caregiving, and prolonged treatment.^27^^,^^28^ Financial hardship can affect adherence and other dimensions of cancer care.^27^^,^^28^ Precision oncology can add costs before treatment through specimen acquisition, genomic testing, confirmatory assays, specialist interpretation, and multidisciplinary review, with coverage and patient responsibility varying across settings. Some genomically matched therapies are standard treatments with established coverage pathways. Others are prescribed off-label, require complex prior authorization, or are available only through clinical trials. Patients may also incur costs associated with additional testing, travel to specialty centers, temporary accommodation, and repeated visits required for trial participation.

In I-PREDICT, treatment selection explicitly incorporated insurance coverage of off-label agents and availability of investigational therapies, demonstrating that molecular matching occurs within, not outside, the realities of the US healthcare financing system.^8^ In the regional MTB study, lack of financial support accounted for another group of patients unable to pursue recommended treatment.^9^ This has an important implication for precision-oncology programs, financial feasibility should be considered at the point of treatment recommendation rather than after the recommendation has been made.

An MTB may conclude that a therapy represents the best molecular match. But if the therapy is unlikely to be covered, requires substantial patient cost sharing, or is available only at a distant trial site, those circumstances influence whether the recommendation constitutes a realistic clinical option. Molecular tumor boards cannot solve drug pricing or insurance policy. They can, however, connect molecular recommendations with pharmacists, financial navigators, prior-authorization specialists, patient-assistance programs, and clinical-trial navigation earlier in the process.

Financial navigation should therefore become part of the precision-oncology pathway rather than an administrative task triggered only after a prescription is denied. The issue is particularly important because financial burden is not distributed evenly. Systematic reviews have identified younger age, lower income, employment disruption, lack of adequate insurance, and other socioeconomic characteristics as risk factors for cancer-related financial hardship.^28^^,^^29^ Thus, a precision-oncology system that produces the same molecular recommendation for 2 patients may nevertheless produce very different treatment opportunities depending on their financial resources and insurance circumstances.

## Disparities can persist after genomic testing

Equity discussions in precision oncology have appropriately emphasized who receives genomic testing. The evidence demonstrates that access remains unequal. A 2026 systematic review of 24 studies found lower utilization of contemporary somatic mutation testing among patients who were older, non-White, socioeconomically disadvantaged, or lacked private insurance.^30^ Large real-world studies have similarly documented disparities. Among patients with metastatic prostate cancer or advanced urothelial carcinoma, Black race, lower socioeconomic status, and Medicaid or Medicare coverage were associated with lower rates of next-generation sequencing.^31^

However, equalizing testing alone will not ensure equitable precision oncology. Disparities can emerge after the test has been performed. A particularly important study of 425 patients with metastatic breast cancer examined this downstream transition.^32^ The proportion of patients with at least 1 actionable alteration detected by cell-free DNA did not differ significantly across racial and ethnic groups, receipt of genotype-matched therapy did.^32^ White patients were significantly more likely to receive matched treatment, and the association persisted after adjustment for tumor subtype, insurance, comorbidity, and distance from the treatment center.^32^

This observation changes the equity question. If patients have comparable rates of actionable findings but different probabilities of receiving the corresponding treatment, then expanding testing alone cannot eliminate the disparity. Potential downstream mechanisms include differences in insurance coverage, referral to specialty centers, access to MTBs, availability of trials, ability to travel, treatment affordability, competing comorbidities, and the distribution of precision-oncology infrastructure across health systems.

The equity objective for precision oncology should therefore expand from equitable testing to equitable translation. Health systems should ask not only whether patients from different populations receive genomic profiling but also whether, among patients with clinically meaningful actionable findings, there are differences in MTB review, referral, treatment initiation, and time to treatment. This downstream perspective is essential because disparities can migrate as technology diffuses. A system can successfully close a gap in genomic testing while leaving intact, or even widening, inequities in access to the treatments those tests identify.

## Molecular tumor boards need to move from interpretation to treatment enablement

Molecular tumor boards have become an important bridge between genomic testing and clinical decision-making. Their multidisciplinary structure brings together oncology, pathology, genomics, genetics, pharmacology, bioinformatics, and other expertise needed to interpret complex findings.^4^^,^^5^ Producing a recommendation, however, should not be the endpoint. The MTB can clarify evidence strength, treatment timing, major eligibility considerations, and realistic treatment or trial options, and it can trigger referral to navigation, pharmacy, financial counseling, genetics, and authorization services. It cannot by itself solve drug pricing, insurance policy, trial geography, or the limited availability of evidence for earlier-line use.

The next generation of MTBs should ask what is required to make the recommendation feasible. For each clinically meaningful recommendation, several questions are immediately relevant (1) Is the recommendation intended for treatment now or for a subsequent line of therapy? (2) What level of evidence supports it? (3) Is the therapy approved and accessible? (4) If it is off-label, what coverage barriers are anticipated? (5) If a clinical trial is recommended, is there an actively recruiting site that the patient can realistically reach? (6) Does the patient meet the major eligibility requirements? (7) If treatment cannot be obtained, what is the next best option? (8) If the recommendation is intended for future use, how will it be reintroduced when it becomes clinically relevant?

The rural academic-community MTB experience illustrates why this expansion matters. Extending molecular expertise into community practice successfully identified actionable alterations and trial opportunities, but identification alone did not overcome distance, transportation, and local trial availability.^21^ Molecular tumor boards should therefore function as treatment-enablement teams within a broader implementation system rather than as molecular-interpretation committees expected to solve every downstream barrier.

## Different barriers require different solutions

The reasons actionable genomic findings do not translate into treatment are heterogeneous and treating them as a single implementation problem obscures the appropriate response. For example, (1) a patient who deteriorates before genomic results become clinically useful points toward the timing of testing; (2) a patient whose recommended trial is hundreds of miles away points toward trial geography and research infrastructure; (3) a patient unable to obtain an off-label targeted therapy points toward coverage and financial access; and (4) a patient who remains on an effective standard therapy despite an actionable finding may represent entirely appropriate cancer care. The practical goal should therefore not be to maximize the proportion of patients receiving matched therapy indiscriminately. It should be to minimize avoidable a loss of clinically meaningful treatment opportunities.

This distinction suggests several priorities for health systems implementing precision oncology: (1) perform comprehensive genomic profiling early enough to inform future treatment planning when clinically indicated; (2) distinguish molecular actionability from immediate clinical actionability; (3) connect MTB recommendations with current and geographically realistic clinical-trial opportunities; and (4) develop academic–community partnerships that extend molecular expertise and trial access beyond tertiary centers. These interventions target the pathway between molecular discovery and actual cancer care rather than simply increasing the volume of genomic testing.

## Conclusion

Precision oncology has made remarkable progress in identifying molecular alterations that can inform cancer treatment. The next implementation challenge is ensuring that clinically meaningful findings can become realistic treatment opportunities without confusing treatment access with treatment effectiveness.

Genomic profiling may occur too late in the disease course, or an earlier profile may become less informative after treatment-related tumor evolution. Relevant trials may exist but be geographically inaccessible. Targeted therapies may encounter insurance or financial barriers, precision-oncology expertise may be concentrated at specialized centers, and disparities can persist even after successful molecular testing. These barriers cannot be solved by more sophisticated sequencing alone.

The next phase of precision oncology requires purposeful initial and, when clinically informative, repeat genomic profiling; realistic trial matching that includes decentralized and community-based access strategies; integration of financial and treatment navigation; stronger connections between academic molecular expertise and community oncology; and explicit attention to disparities downstream from testing. An actionable genomic alteration represents biological possibility. The goal of precision oncology should be to ensure that, when clinically appropriate, it also represents a realistic treatment opportunity while evaluating clinical benefit separately rather than assuming or guaranteeing it.

## Data Availability

No data were generated or analyzed in support of this manuscript.

## References

[1] Shah NM , Meric-Bernstam F. The present and future of precision oncology and tumor-agnostic therapeutic approaches. Oncologist. 2025;30:oyaf152. 10.1093/oncolo/oyaf152 PMC12204399 40536268

[2] Dalton WB , Forde PM, Kang H, et al Personalized medicine in the oncology clinic: implementation and outcomes of the Johns Hopkins molecular tumor board. JCO Precis Oncol. 2017;1-19. 10.1200/PO.16.00046 PMC6039131 30003184

[3] Chornokur G , Dalton K, Borysova ME, Kumar NB. Disparities at presentation, diagnosis, treatment, and survival in African American men, affected by prostate cancer. Prostate. 2011;71:985-997. 10.1002/pros.21314 PMC3083484 21541975

[4] Tsimberidou AM , Kahle M, Vo HH, et al Molecular tumour boards—current and future considerations for precision oncology. Nat Rev Clin Oncol. 2023;20:843-863. 10.1038/s41571-023-00824-4 37845306

[5] Larson KL , Huang B, Weiss HL, et al Clinical outcomes of molecular tumor boards: a systematic review. JCO Precis Oncol. 2021;5:PO.20.00495. 10.1200/po.20.00495 PMC8277300 34632252

[6] Massard C , Michiels S, Ferté C, et al High-throughput genomics and clinical outcome in hard-to-treat advanced cancers: results of the MOSCATO 01 Trial. Cancer Discov. 2017;7:586-595. 10.1158/2159-8290.Cd-16-1396 28365644

[7] Rodon J , Soria J-C, Berger R, et al Genomic and transcriptomic profiling expands precision cancer medicine: the WINTHER trial. Nat Med. 2019;25:751-758. 10.1038/s41591-019-0424-4 PMC6599610 31011205

[8] Sicklick JK , Kato S, Okamura R, et al Molecular profiling of cancer patients enables personalized combination therapy: the I-PREDICT study. Nat Med. 2019;25:744-750. 10.1038/s41591-019-0407-5 PMC6553618 31011206

[9] Peh KH , Przybylski DJ, Fallon MJ, et al Clinical utility of a regional precision medicine molecular tumor board and challenges to implementation. J Oncol Pharm Pract. 2023;29:1094-1102. 10.1177/10781552221091282 35369813

[10] Fröhlich T , Lehmann U, Reineke-Plaaß T, et al From recommendation to implementation: clinical outcomes of a single-center molecular tumor board. Target Oncol. 2026;21:593-608. 10.1007/s11523-026-01231-2 PMC13427782 42467374

[11] Mateo J , Chakravarty D, Dienstmann R, et al A framework to rank genomic alterations as targets for cancer precision medicine: the ESMO Scale for Clinical Actionability of Molecular Targets (ESCAT). Ann Oncol. 2018;29:1895-1902. 10.1093/annonc/mdy263 PMC6158764 30137196

[12] Lin FP , Thavaneswaran S, Grady JP, et al; Australian Molecular Screening and Therapeutic Program Investigators and Contributors. Genomic therapy matching in rare and refractory cancers. JAMA Oncol. 2026;12:458-467. 10.1001/jamaoncol.2026.0127 PMC12964253 41784981

[13] Giacomini P , Valenti F, Allegretti M, et al The molecular tumor board of the Regina Elena National Cancer Institute: from accrual to treatment in real-world. J Transl Med. 2023;21:725. 10.1186/s12967-023-04595-5 PMC10577953 37845764

[14] Tarawneh TS , Rodepeter FR, Teply-Szymanski J, et al Combined focused next-generation sequencing assays to guide precision oncology in solid tumors: a retrospective analysis from an Institutional Molecular Tumor Board. Cancers. 2022;14:4430. 10.3390/cancers14184430 PMC9496918 36139590

[15] Bryce AH , Egan JB, Borad MJ, et al Experience with Precision Genomics And Tumor Board, indicates frequent target identification, but barriers to delivery. Oncotarget. 2017;8:27145-27154. 10.18632/oncotarget.16057 PMC5432324 28423702

[16] Ferreira-Gonzalez A , Ko G, Fusco N, et al Barriers and facilitators to next-generation sequencing use in United States oncology settings: a systematic review. Future Oncol. 2024;20:2765-2777. 10.1080/14796694.2024.2390821 PMC11572137 39316553

[17] Tamborero D , Dienstmann R, Rachid MH, et al; Cancer Core Europe Consortium. The molecular tumor board Portal supports clinical decisions and automated reporting for precision oncology. Nat Cancer. 2022;3:251-261. 10.1038/s43018-022-00332-x PMC8882467 35221333

[18] U.S. Food & Drug Administration, Oncology Center of Excellence. Project FrontRunner: advancing development of oncology therapies to the early clinical setting. Accessed September 25, 2026. https://www.fda.gov/about-fda/oncology-center-excellence/project-frontrunner

[19] Chakravarty D , Johnson A, Sklar J, et al Somatic genomic testing in patients with metastatic or advanced cancer: ASCO provisional clinical opinion. J Clin Oncol. 2022;40:1231-1258. 10.1200/jco.21.02767 35175857

[20] Beckman RA , Loeb LA. Rare mutations in cancer drug resistance and implications for therapy. Clin Pharmacol Ther. 2020;108:437-439. 10.1002/cpt.1938 PMC7484911 32648584

[21] Kumar A , Owen JR, Sloat NT, et al Expansion of an academic molecular tumor board to enhance access to biomarker-driven trials and therapies in the rural southeastern United States. Curr Oncol. 2024;31:7244-7257. 10.3390/curroncol31110534 PMC11593073 39590164

[22] Uehara Y , Koyama T, Katsuya Y, et al Travel time and distance and participation in precision oncology trials at the National Cancer Center Hospital. JAMA Netw Open. 2023;6:e2333188. 10.1001/jamanetworkopen.2023.33188 PMC10504617 37713200

[23] Kirkwood MK , Schenkel C, Hinshaw DC, et al State of geographic access to cancer treatment trials in the United States: are studies located where patients live? JCO Oncol Pract. 2025;21:427-437. 10.1200/op.24.00261 PMC11925346 39356976

[24] Gueguen L , Olgiati L, Brutti-Mairesse C, et al A prospective pragmatic evaluation of automatic trial matching tools in a molecular tumor board. NPJ Precis Oncol. 2025;9:28. 10.1038/s41698-025-00806-y PMC11772588 39870746

[25] Pishvaian MJ , Blais EM, Bender RJ, et al A virtual molecular tumor board to improve efficiency and scalability of delivering precision oncology to physicians and their patients. JAMIA Open. 2019;2:505-515. 10.1093/jamiaopen/ooz045 PMC6994017 32025647

[26] Ghadessi M , Di J, Wang C, et al Decentralized clinical trials and rare diseases: a Drug Information Association Innovative Design Scientific Working Group (DIA-IDSWG) perspective. Orphanet J Rare Dis. 2023;18:79. 10.1186/s13023-023-02693-7 PMC10088572 37041605

[27] Zafar SY. Financial toxicity of cancer care: it’s time to intervene. J Natl Cancer Inst. 2016;108:djv370. 10.1093/jnci/djv370 26657334

[28] Smith GL , Lopez-Olivo MA, Advani PG, et al Financial burdens of cancer treatment: a systematic review of risk factors and outcomes. J Natl Compr Canc Netw. 2019;17:1184-1192. 10.6004/jnccn.2019.7305 PMC7370695 31590147

[29] Samaha NL , Mady LJ, Armache M, et al Screening for financial toxicity among patients with cancer: a systematic review. J Am Coll Radiol. 2024;21:1380-1397. 10.1016/j.jacr.2024.04.024 38762031

[30] Rae S , Baldwin A, Lagonera MJ, et al Are there socio-demographic inequalities in the utilisation of tumour and ctDNA somatic mutation testing in solid tumours? A systematic review. Cancer Med. 2026;15:e71668. 10.1002/cam4.71668 PMC13093295 41830052

[31] Hage Chehade C , Jo Y, Gebrael G, et al Trends and disparities in next-generation sequencing in metastatic prostate and urothelial cancers. JAMA Netw Open. 2024;7:e2423186. 10.1001/jamanetworkopen.2024.23186 PMC11258596 39023888

[32] Sood R , Niemierko A, Ryan L, et al Impact of race/ethnicity on clinical and genomic characteristics, trial participation, and genotype-matched therapy among patients with metastatic breast cancer. Clin Cancer Res. 2025;31:1315-1322. 10.1158/1078-0432.Ccr-24-2825 39869305

